# Characterization of the Coating Layers Deposited onto Curved Surfaces Using a Novel Multi-Nozzle Extrusion Printer

**DOI:** 10.3390/mi16050505

**Published:** 2025-04-26

**Authors:** Ramses Seferino Trigo Torres, Lawrence Kulinsky, Arash Kheradvar

**Affiliations:** 1 Department of Biomedical Engineering, University of California-Irvine, Irvine, CA 92697, USA; rtrigoto@uci.edu; 2 Department of Mechanical and Aerospace Engineering, University of California-Irvine, Irvine, CA 92697, USA

**Keywords:** 3D printing, bioprinting, extrusion, prototyping, coating, tissue engineering

## Abstract

Over the past two decades, additive manufacturing has advanced significantly, enabling rapid fabrication of functional components across various applications. In medical devices, it has been used for prototyping, prosthetics, drug delivery platforms, and more recently, tissue scaffolding. However, current technologies face challenges, particularly in depositing conformal layers over curved surfaces. This study introduces a novel multi-nozzle extrusion printer concept designed to deposit soft gel layers onto curved surfaces. A custom clearance locking mechanism enhances the printer’s ability to achieve conformal coatings on both flat and curved substrates. We investigate key deposition parameters, including displacement volume and nozzle configuration, while comparing two deposition sequences: “Press and Express” and “Express and Press”. Our results demonstrate that the “Express and Press” technique yields more uniform, merged conformal layers than the “Press and Express” method. This technology holds promise for further refinement and potential applications in tissue engineering.

## 1. Introduction

Over the past two decades, significant strides in additive manufacturing have propelled its widespread application across various fields, notably in medical devices [1,2]. The latest advancements in additive manufacturing technologies can address some of the most complex biofabrication problems in tissue engineering and regenerative medicine [3,4]. To accomplish specific fabrication goals, various technologies should be utilized simultaneously or in sequence.

For instance, Fused Filament Fabrication (FFF), also known as Fused Deposit Manufacturing (FDM), provides a cost-effective but low-resolution platform suitable for early prototyping with sub-millimeter-sized features [5,6,7,8]. Selective Laser Sintering (SLS), utilizing a powder bed technology, operates a powerful laser to fuse particles into layers of predetermined geometry. SLS enables the construction of features with complex internal and external geometries, commonly used in bone tissue engineering [6,9]. Another technology involving the curing of photosensitive resin, as seen in stereolithography (SLA), is applicable in various contexts such as the production of microfluidic platforms [10,11].

Despite the appealing features of additive manufacturing technologies, significant challenges persist in depositing soft layers onto surfaces with intricate topologies, such as the concave surfaces of tissue-engineered heart valves (TEHVs) [12,13,14,15,16]. The delicacy and sensitivity of biological layers containing living cells add further complexity, requiring meticulous control of sheer stresses and maintaining processing temperatures below 37 °C to preserve cell viability [17]. Extrusion-based 3D printing, a popular bioprinting methodology, addresses some of these challenges by allowing for precise printing of complex geometries without the use of high temperatures, relying on chemical or photoactivated curing steps for cross-linking the printed soft material [18]. Hydrogels, widely used in extrusion-based 3D printing, serve as effective materials, either for creating scaffolds to which cell layers are added later or for directly depositing cell-laden gels as “bio-inks” [18,19,20,21,22,23,24,25].

Nevertheless, numerous obstacles, including concerns about cell viability, adherence, and a narrow range of printing conditions, still demand resolution [18,19,20,21]. Additionally, existing printing platforms lack the capability for conformal coating on contoured substrates without tilting the substrate [19].

Two notable strategies have overcome the obstacles outlined to advance the printing of soft materials on curved surfaces: robotic arm-based printing [26,27,28] and multi-material multi-nozzle adaptive 3D printing [29]. Both approaches necessitate an initial surface scan to create a three-dimensional profile of the surface intended for coating. This geometry then serves as the foundation for generating non-planar toolpath traversal algorithms. In certain situations, the volumetric displacement of the ink may be altered during deposition based on the nozzle’s specific position, thereby causing variations in layer thickness [27,28]. Deposition speed can also be enhanced by employing a line of multiple nozzles instead of a single nozzle [29]. However, these approaches come with some limitations. For example, they both rely on surface profiles generated through prior scanning and tend to be costly.

This study introduces a novel multi-nozzle bioprinter capable of coating conformal layers (MBCCL) of gel over geometrically flat, convex, or concave surfaces, enabled by the computer numerical control (CNC) platform. The MBCCL allows gels to be displaced through multiple small nozzles, with the ability to individually control the amount of displaced gel in each nozzle’s array. The current study outlines the MBCCL’s principles of conformal coating deposition and presents experimental results.

Mathematical modeling relating layer thickness and topology to deposition parameters, such as a syringe’s plunger displacement and gel droplet size, is also discussed. This soft matter extrusion printer concept serves as an enabling technology that, with further development, has the potential to facilitate the conformal coating of cell-laden hydrogels over curved surfaces, ushering in new advancements in tissue engineering. The approach presented here eliminates the need for prior surface scans and utilizes inexpensive components, making it potentially suitable for widespread adoption.

## 2. Materials and Methods

### 2.1. Multi-Nozzle Extrusion 3D Printer

Here, we describe the design and development of a multi-nozzle extrusion printer.

#### 2.1.1. Multi-Nozzle Attachment

The initial prototype of the extrusion 3D printer featured a 2 × 2 square nozzle array (McMaster-Carr, Santa Fe Springs, CA, USA, 0.042″ Stainless Steel Tubing) with a center-to-center distance of 2.5 mm, connected via clear plastic tubing (McMaster-Carr, Santa Fe Springs, CA, USA, 1883T3) to four syringes (Monoject, Cardinal Health, Dublin, OH, USA, 60 mL Luer Lock Syringe) filled with green aloe vera gel (density: 1.2 g/cm^3^; viscosity: 4.2 cP at 25 °C). The syringe plungers were actuated using a stepper motor (Motech Motor, Guangzhou, China, MT-1704-HS168A-0B).

To achieve enhanced gel feeding control, the second prototype was fabricated to include the expanded 4 × 4 nozzle array (McMaster-Carr, Santa Fe Springs, CA, USA, 0.042″ Stainless Steel Tubing), replicating the independent feeding mechanism of the initial prototype but with smaller diameter syringes (Care Touch, Brooklyn, NY, USA, 1 mL Luer Slip Tip Syringe). Additionally, a standalone extrusion printer with a single nozzle (McMaster-Carr, Santa Fe Springs, CA, USA, 0.042″ Stainless Steel Tubing) and syringe (Care Touch, Brooklyn, NY, USA, 1 mL Luer Slip Tip Syringe) was developed to analyze droplet behavior.

Computer Numeric Control of the Extrusion Printer: The extrusion printer setup employs a multi-nozzle attachment with a locking system integrated into a CNC-controlled platform. The CNC setup comprises T-slotted framing (McMaster-Carr, Santa Fe Springs, CA, USA, 47065T101) with two stepper motors for the *X*-axis, one on each side, one stepper motor for the *Y*-axis, and one for the *Z*-axis (Motech Motor, Guangzhou, China, MT-1704-HS168A-0B). Positional control is executed through an Arduino UNO (Arduino UNO Rev3, Scarmagno, Italy) with a CNC expansion board (CNC Shield Board, Lewes, DE, USA) and four stepper motor drivers (Pololu Robotics and Electronics, Las Vegas, NV, USA, A4988 Stepper Motor Driver).

#### 2.1.2. Printing Process

**Figure 1:**
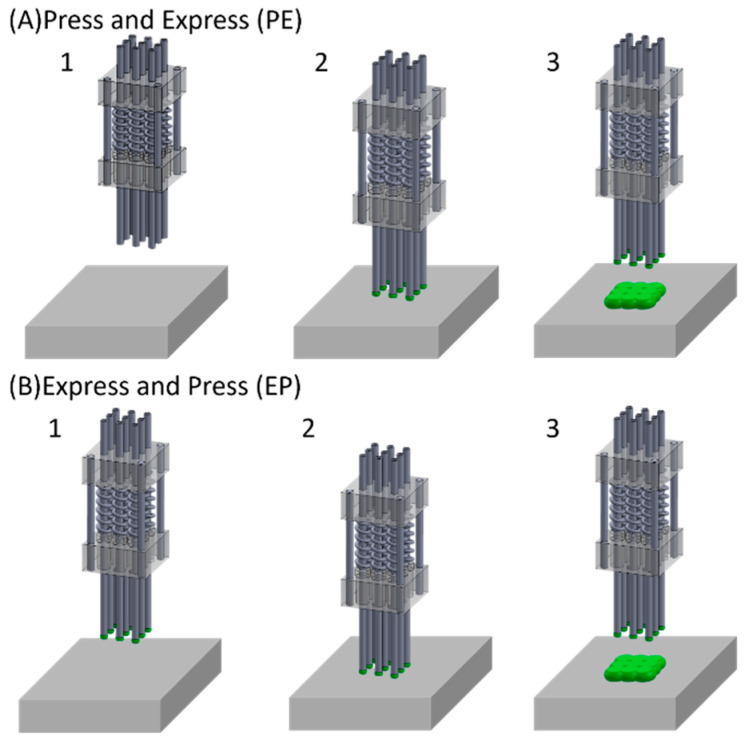
Schematics for the “Press and Express” and “Express and Press” deposition techniques. (**A1**) Positioning f a multi-nozzle attachment over the surface. (**A2**) Lowering the nozzle array onto the surface, expressing gel through the nozzles. (**A3**) Raising the multi-nozzle attachment, separating it from the surface. (**B1**) Positioning the multi-nozzle attachment over the surface, expressing gel from the nozzles to create suspended droplets. (**B2**) Lowering the multi-nozzle attachment onto the surface. (**B3**) Lifting the multi-nozzle attachment from the surface.

Press and Express vs. Express and Press Deposition Techniques: Two deposition techniques were explored: the “Press and Express” (PE) technique involves pressing the extrusion printer head onto the surface before expressing the gel, while the “Express and Press” (EP) technique reverses the sequence. The schematics of the PE and EP deposition techniques are presented in Figure 1. Experimental comparisons were conducted to evaluate these approaches.

Clearance Locking System: An inventive feature integrated into the extrusion printer is its clearance locking system, enabling the conformal coating of curved surfaces using the multi-nozzle array. The effectiveness of the “Express and Press” technique hinges on maintaining a consistent clearance (gap) between the surface intended for gel coating and the nozzle edge.

For curved surfaces, especially convex ones, achieving this constant clearance poses a challenge. The nozzles must be secured in a manner that aligns with the surface’s topology, ensuring an identical distance between all nozzles in the array and the curved surface. In instances where all nozzles protrude the same distance from the holder, establishing a constant clearance becomes intricate when dealing with curved surfaces.

**Figure 2:**
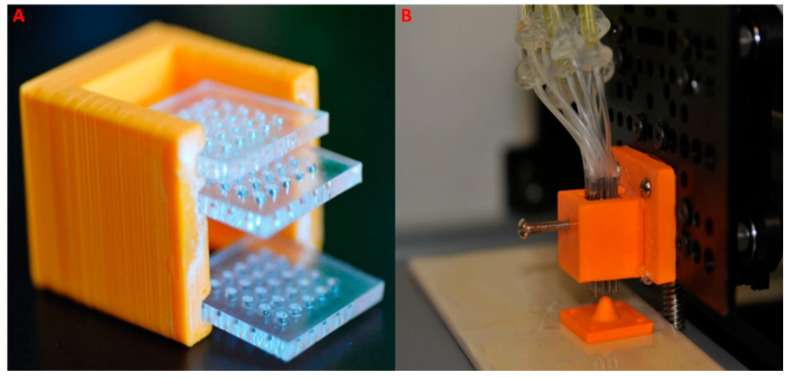
The extrusion printer head’s locking mechanism. (**A**) The internal structure of the extrusion head’s locking mechanism. The upper and lower plates secure the nozzles in place, while the middle plate is pushed by a screw to lock the nozzles in the desired position. All three plates are extended out of the holder to illustrate their design. (**B**) The assembled extrusion printer head, where the nozzles protrude to varying extents to conform to a curved surface (the orange cone below the array). The locking screw is visible on the left side of the extrusion printer’s nozzle array holder.

To overcome this challenge, a locking plate, illustrated in Figure 2, was introduced. This locking plate incorporates a screw that exerts lateral pressure on the middle plate, thereby restricting the vertical movement of the nozzles within the array. When the spring-loaded nozzle array is lowered onto the curved surface, the locking screw engages, pushing over the middle plate. The transverse force applied to the middle plate constrains the movement of individual nozzles. Upon raising the nozzle array off the surface, a constant distance between the individual nozzles and the curved surface is maintained. This innovative solution ensures the reliable implementation of the “Express and Press” deposition process for conformal coating on curved surfaces.

**Figure 3:**
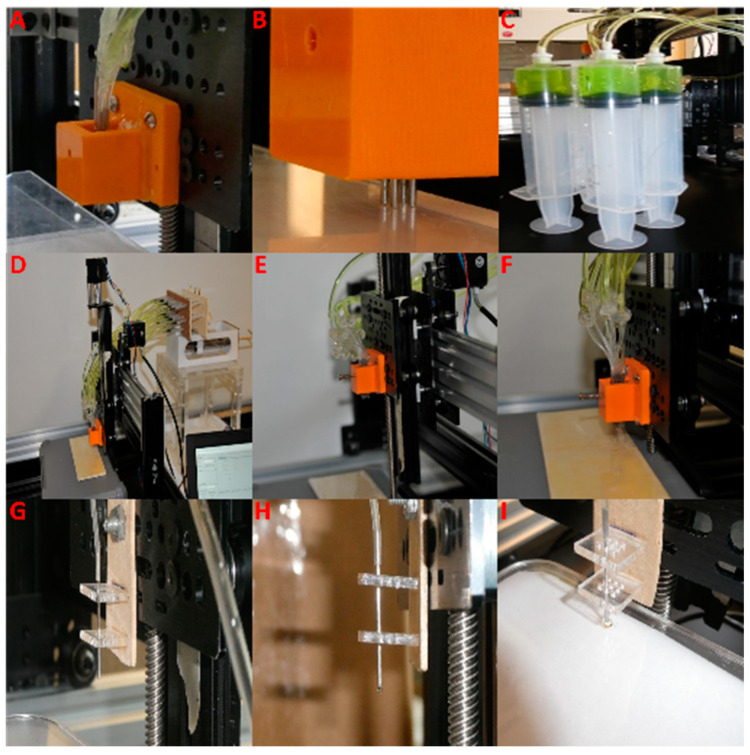
Multi-nozzle attachments. (**A**) A 2 × 2 array multi-nozzle attachment with individual feeding for each nozzle. (**B**) A 2 × 2 nozzle array multi-nozzle attachment with individual feeding for each nozzle. (**C**) A 2 × 2 nozzle array multi-nozzle attachment where each nozzle is connected to a 60 mL syringe. (**D**–**F**) A second prototype featuring a 4 × 4 nozzle array with individual feeding for each nozzle. (**G**–**I**) A single-nozzle setup of the extrusion printer used for characterizing gel deposition parameters.

Independent Gel Flow: Experimental runs involving a single syringe connected to both 4- and 16-nozzle arrays revealed an uneven distribution of gel due to disparate pressure within the nozzle array. To address non-uniform gel distribution, a gel distributor system was devised, connecting each nozzle to an individual gel-filled syringe (refer to Figure 3). This innovative approach significantly enhanced the deposition process, resulting in a more uniform gel layer. The system demonstrated superior control over individual droplets expressed from the nozzles, surpassing the limitations observed in the single-syringe deposition technique. Further details and comparisons are elaborated in the subsequent “Results” section.

Maximum Droplet Volume for Controlled Deposition: As larger droplets emerge from the nozzles, a critical point is reached where the droplets’ weight surpasses the surface tension force, causing premature separation before the nozzle array is lowered to the surface.

Conversely, when droplets are exceedingly small, barely wider than the nozzle diameter, suboptimal outcomes ensue. During the lowering of the nozzle array onto the surface, these small droplets fail to merge into a single conformal layer, persisting as an array of individual gel droplets. To evaluate the control of deposition relative to droplet size within a fixed time-lapse (60 s), individual gel depositions of 1500 µL, 1000 µL, and 500 µL were conducted. Larger gel volumes, however, led to irregular deformation and a loss of spherical droplet profiles. Remarkably, 500 µL depositions resulted in the preservation of spherical droplet shapes.

### 2.2. Mathematical Modeling

The size of the droplet is influenced by various factors, including surface tension, viscosity, nozzle size, the density of the soft matter, and the rheological properties of the gel—especially if it contains secondary-phase dispersions like biological cells. Additionally, temperature plays a role by affecting viscosity and surface tension, while other factors such as applied pressure, the clearance between the nozzle and the surface, and other controlling variables also contribute. Although creating a fully validated parametric model is impractical, we have chosen to present a first-order approximation for the size of the deposited droplet.

A phenomenological mathematical model has been developed to elucidate the height of the conformal layer as a function of deposition parameters specific to the “Express and Press” technique.

The model articulates how the displacement of material within a syringe influences both the approximate droplet size and the resultant height of the merged layer. Considered at room temperature, this model is specific to a 16-nozzle extrusion printer (4 × 4 array) with 2.5 mm spacing between nozzle centers. Notably, this model is readily adaptable to other nozzle arrays that are equidistant and positioned in a square grid configuration.

In deriving the height of the deposited layer, several simplifying assumptions were embraced:

1. The gel behaves as an incompressible fluid, with no retraction into the nozzles once expressed.
2. The droplets’ geometry, resembling a truncated sphere with a diameter 1.5 times greater than the corresponding nozzle’s diameter (d_D_), is deemed stable, validated by experimental observations (as depicted in Supplementary Figure S1).
3. Homogeneous merging characterizes all deposited droplets.

Consequently, the total volume expressed from the 16 nozzles equates to 16 X_S_, where X_S_ represents the volume of gel displaced by each syringe. The nozzles within the array are arranged in a square grid with a side length denoted as L_m_, measured from the center to the center of the corner nozzles on each side of the grid. Given this assumption, the gel spreads in a square layer with a size equal to L_m_ + 1.5d_D_. Thus, the predicted height, H_L_, of the deposited gel layer is determined by dividing the expressed gel volume by the lateral area covered by the gel.

(1) $$H_{L}=\frac{16X_{S}}{{\left(L_{m}+1.5d_{D}\right)}^{2}}$$

Additionally, an equation for determining the deposited volume of gel (D_g_) has been devised. This equation considers factors such as fluid compressibility (қ), system leaks (L_g_), gel retraction into the nozzles post-droplet deposition (T_g_), the number of nozzles (*n*_#_), and the volume per nozzle (V_n_). The formulated equation is presented below:

(2) $$D_{g}=(V_{n}*n_{\#})-(қ+L_{g}+T_{g})$$

This equation serves to provide a comprehensive understanding of the gel deposition process, considering various contributing factors that influence the accuracy and reliability of the outcomes. An approximation of Equation (2) is shown in Table 1.

In addition to Equation (2), a third equation has been formulated to validate the conformal coating of the presented samples. This equation facilitates the calculation of gel layer height at any specified point along the sample:

(3) $$Y_{c}=Y_{s}+h_{g}$$

In this context, Y_c_ denotes the measured height of the sample, Y_s_ represents the original height of the sample without the gel layer, and hg signifies the height of the gel layer.

## 3. Results and Discussions

### 3.1. Characterization of Single-Droplet Deposition on a Flat Surface

To comprehensively understand gel deposition using both PE and EP approaches before incorporating the nozzle array, we conducted and evaluated deposition via a single nozzle using a commercial aloe vera gel to mimic the behavior of gel-like materials within the system, along with type 1 rat tail collagen and thiolated hyaluronic acid, two widely used components in bio-ink formulations and 3D hydrogel fabrication for bioprinting.

Due to the low viscosity of the collagen and hyaluronic acid in their initial stages before hydrogel formation, PE deposition was only tested for the aloe vera gel. The nozzle was initially lowered over the target surface, followed by the expression of the material through the nozzle. Subsequently, the nozzle was disengaged from the surface, and the coated area was visually examined. Image analysis, performed with ImageJ software (National Institutes of Health, open source, version 1.54p, Bethesda, MD, USA), quantified the diameter of the coated area and the final droplet area using both top and side views (refer to Supplementary Figure S1A). The droplet exhibited a slightly elongated oval shape, with a measured area of 7.77 mm^2^, as determined by the circumference analysis.

It is crucial to choose springs with appropriate stiffness for the type of gel being deposited using the PE technique. If spring stiffness is too high, it may be difficult to express the gel, whereas springs that are too soft could cause the gel to spread excessively. Achieving the right balance requires experimental determination, taking into account the spring stiffness, gel properties, and applied pressure to ensure precise and controlled “Press and Express” deposition.

Subsequent to the PE test, the EP assessment was conducted. During the EP evaluation, gel expression occurred with the nozzle tip positioned 1 mm above the flat surface. Instantaneous photographs of the gel droplet were captured. The pre-deposition images of the droplet were subjected to analysis using ImageJ software. Notably, the suspended droplet exhibited a non-spherical form, as illustrated in Supplementary Figure S1B. To measure its size, a mesh comprising six horizontal and seven vertical lines was overlaid on the droplet image, and the resulting grid measurements are detailed in Supplementary Table S1.

Once the droplet was expressed over the surface, a second image (side view presented in Supplementary Figure S1C) was taken before separating the nozzle from the droplet. This image underwent analysis to determine the size of the deposited droplet.

Following deposition, the lateral dimensions of the deposited droplet were greatly expanded compared with the suspended droplet as the nozzle pushed the droplet down and shear stresses facilitated the spreading of the droplet on the surface. While the suspended droplet had a mean diameter of 1.24 mm (side view), as it was deposited over the surface by using the EP technique, the top view of the deposited droplet presented a nearly circular shape with a mean diameter of 3.41 mm. After the nozzle was lifted and the droplet rested over the surface (see Supplementary Figure S1D), its two major axes, identified on Supplementary Figure S1D as 1 and 2, respectively, were 5.07 mm and 4.88 mm and the calculated area covered by the deposited gel droplet was 15.65 mm^2^.

Even though collagen and hyaluronic acid solutions presented a lower viscosity compared to the aloe vera gel which made PE deposition not possible, EP deposition was made possible due to the close distance between the nozzle and the printing surface. Likewise with the gel, we were able to generate a droplet that held its shape for several seconds before the nozzles were lowered toward the surface to be coated. The behavior of both biomaterials was similar to the behavior of the aloe vera gel, as the droplets stayed attached to the nozzle until some form of vibration was applied, with the only difference being that the shapes of collagen and hyaluronic acid were spherical compared to the non-spherical shape presented by the gel, as shown in Supplementary Figure S1B.

Since both droplets were shaped closely to a spherical shape, 12 lines were drawn vertically and horizontally, 6 lines each, to measure their size. The resulting grid measurements are detailed in Supplementary Table S2.

Likewise with the aloe vera gel, both collagen and hyaluronic acid droplets were greatly expanded compared with the suspended droplet. While the suspended droplet had a mean diameter of 1.70 mm for collagen and 1.49 mm for hyaluronic acid, the top view of both droplets presented a circular shape with a mean diameter of 4.95 mm and 2.35 mm, respectively. After the droplets were deposited over the surface and the nozzle was lifted (Supplementary Figure S2B,E), their major axes identified as 1 and 2 in both figures were 4.62 mm and 5.28 mm, respectively, for collagen and 2.23 mm and 2.47 mm, respectively, for hyaluronic acid. With these axes, we were able to calculate an estimated covered area of 19.07 mm^2^ for collagen and 4.53 mm^2^ for hyaluronic acid.

Utilizing the EP deposition technique enables the deposition of gel droplets, extending beyond the cross-sectional area of individual nozzles. Consequently, the lateral dimensions of each deposited droplet surpass the distances between the nozzles in the array. This phenomenon facilitates the merging of adjacent droplets on the surface, giving rise to the formation of a continuous and conformal layer.

### 3.2. Gel Deposition over a Flat Surface Using a 4-Nozzle Array

Our investigation into gel deposition involved a 4-nozzle array, with each nozzle individually connected to a syringe. The deposition process commenced by lowering the extrusion printer head onto the surface, causing the nozzle array’s springs to compress by several millimeters. The extrusion printer head was incrementally raised in 0.5 mm steps until the nozzle tips lightly made contact with the surface (refer to Figure 1B).

For both PE and EP gel deposition, three gel displacement volumes—500 µL, 1000 µL, and 1500 µL—were used. Each displacement volume experiment was conducted three times. In the case of EP deposition experiments, the droplets were generated at a clearance of 0.5 mm (the gap between the nozzle tip and the surface). Deposition results are presented in Supplementary Figure S3. Further details outlining the characteristics of the gel layers produced by using the PE and EP techniques can be found in Supplementary Figure S4.

In the case of the PE technique, a consistent pattern was observed during and after deposition across all three different gel volumes. During displacement, notable back pressure occurred within the nozzles, causing the syringe to retract, thereby hindering the deposition of gel for the 500 µL and 1000 µL volume displacements. Conversely, in the case of the 1500 µL volume displacement, excessively high pressure within the system led to the gel being forcibly expelled through the nozzles and other areas, resulting in leaks. The deposited gel tended to accumulate and form irregular shapes post-deposition, as evident in Supplementary Figure S4A, in contrast to the results of EP deposition described subsequently (Supplementary Figure S4B,C).

In the process of EP deposition, when using volumes exceeding 1000 µL, the weight of the gel droplets overcame surface tension. Consequently, for these substantial volumetric displacements, there was no intermediate step where the drops were suspended from the tips of the nozzles; instead, the gel was promptly deposited directly onto the surface. Supplementary Table S3 illustrates the surface area covered by the gel deposited using three distinct volumetric displacements for the 4-nozzle array.

Upon measuring the three distinct cases, a noticeable variance of approximately 69 mm^2^ was identified between the 500 µL and 1000 µL depositions, and approximately 60 mm^2^ between the 1000 µL and 1500 µL depositions. This variation in deposited volume is likely attributed to the gel’s compressibility, some degree of gel retraction into the nozzles post-deposition, and potential gel leakage within the system. Additionally, it was observed that the 1000 µL and 1500 µL depositions tended to accumulate gel to a height approximately 2 mm greater in the center of the deposited gel than at its edges.

### 3.3. The Deposition of Gel onto a Flat Surface Employing a 16-Nozzle Array

We designed and fabricated a sixteen-nozzle array setup to enhance the uniformity of gel deposition over a larger area. Instead of the previously used 60 mL syringes, we utilized 16 smaller 1 mL syringes, each connected to an individual nozzle in the array. The EP gel deposition technique was employed for volumetric depositions of 100 µL, 200 µL, and 300 µL, maintaining a clearance of 0.5 mm between the nozzle array and the surface.

Supplementary Figure S4A–C present the outcomes of EP deposition with volumetric depositions of 100 µL, 200 µL, and 300 µL per nozzle, respectively. The corresponding surface areas covered by the gel in these depositions are detailed in Table 1, where the longitudinal diameter is parallel to the *X*-axis, and the transverse diameter is perpendicular to the *X*-axis, as illustrated in Supplementary Figure S4D–F.

In addition, the height of the deposited gel was measured to elucidate the topology of the layer. Side views of the deposited gel samples are presented in Supplementary Figure S4D–F. The average height of each deposited layer was calculated as the average of the values along the vertical white lines outlined in the photographs. The results of these measurements are summarized in Table 1.

Table 2 provides a summary, including a comparison of the calculated gel volume displaced versus the desired volume and gel loss per sample, as described in Equation (2). Notably, there is significant variation among the three samples concerning both the topology of the deposited gel layer and the actual volume deposited. Addressing this variation and improving flow control for the gel will be the focus of future studies aimed at reducing sample-to-sample variability.

Moreover, upon analyzing the data in Table 1 and Table 2, it becomes apparent that the lateral spread of the deposited gel exceeds the dimensions predicted by the “rectangular prism” model outlined in Equation (1). This model assumes that the deposited gel layer will closely match the area of the nozzle array. However, as indicated in Table 1, for deposited volumes of 100, 200, and 300 microliters, the gel deposits assume an ellipsoidal shape rather than a square one as modeled. The average diameters of these gel deposits are approximately 13, 20, and 27 mm, respectively. Each of these diameters surpasses the value of L_m_ + 1.5d, which, for a 16-nozzle array, is equal to 9 mm.

Additionally, the approximate areas of the gel deposits, measuring 142, 340, and 528 mm^2^, are considerably larger than the square gel deposit with an area of 81 mm^2^ predicted by Equation (1). The data in Table 1 and Table 2 further reveal that as the displaced volume of the gel increases, so does the lateral spread. This contradicts the implication of Equation (1), which suggests that only the height of the gel deposit would increase with an elevated displaced gel volume while keeping the footprint constant.

In light of these observations, it is evident that the model described by Equation (1) is not well suited for the extrusion printer. The specific lateral spread and heights of the deposited layers appear to be contingent on the physical properties of the materials under study.

### 3.4. The Deposition of Gel on a Curved Surface Employing a 16-Nozzle Array

**Figure 4:**
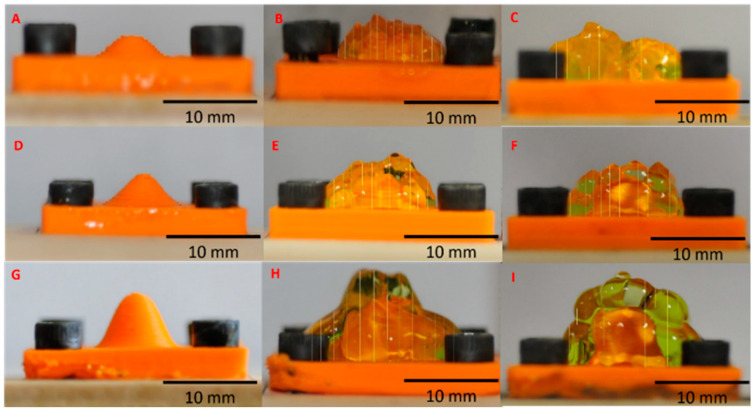
Surface coating utilizing a 16-nozzle array with the EP approach on curved testbed. (**A**) An uncoated 2 mm sample. (**B**) A coated 2 mm sample with 12 vertical lines for layer height assessment using uniform displacement (UD). (**C**) A coated 2 mm sample with 12 vertical lines for layer height assessment using selective displacement (SD). (**D**) An uncoated 4 mm sample. (**E**) A coated 4 mm sample with 12 vertical lines for layer height assessment using uniform displacement (UD). (**F**) A coated 4 mm sample with 12 vertical lines for layer height assessment using selective displacement (SD). (**G**) An uncoated 6 mm sample. (**H**) A coated 6 mm sample with 12 vertical lines for layer height assessment using uniform displacement (UD). (**I**) A coated 6 mm sample with 12 vertical lines for layer height assessment using selective displacement (SD).

Experiments involving the deposition of gel layer using a 16-nozzle arrangement were conducted on three convex test surfaces. These testbeds comprised a 3D printed platform measuring 25 mm × 25 mm × 3.30 mm, featuring sinusoidal shapes with heights of 2 mm, 4 mm, and 6 mm, as illustrated in Figure 4A, Figure 4D, and Figure 4G, respectively. The chosen heights align with the maximum vertical travel distance of the nozzles in the tested prototypes of the extrusion 3D printer, which is approximately 6 mm.

Based on the presented results, a volume displacement of 300 µL was chosen for each nozzle. A consistent 1 mm clearance between the nozzles and the surface was maintained throughout all experiments. The test surfaces were affixed to a Balsa Wood sheet (Walmart, Balsa Wood sheets for DIY Projects) using hex head fasteners (McMaster-Carr) for stability.

The extrusion printer was positioned centrally above each 3D printed sample surface. The extrusion printer head was gradually lowered until all 16 nozzles made contact with the surface, causing the springs of each nozzle to compress. Subsequently, the locking screw was engaged to secure the nozzles in place, resulting in the negative (concave) imprint of the convex surface by the nozzle array. The printer head was then elevated until a 1 mm clearance was attained and 300 µL of gel was displaced. The deposition process was finalized by raising the printer nozzle head.

The outcomes of gel layer deposition on three curved surfaces with varying curvatures are depicted in Figure 4B,E,H. In all three samples, the gel layer seamlessly merged, providing uninterrupted coverage. For the smaller 2 mm tall “hill,” both the hill and the surrounding platform were entirely submerged under the uniformly deposited layer. Conversely, for taller features, the gel layer conformed to the overall shape of the underlying surface, resulting in a protruding gel structure above the corresponding hills.

Due to the independent feeding capacity of our extrusion printer head’s nozzles, we had the flexibility to choose a subset of nozzles that could displace a different amount of gel compared to the remaining nozzles. This technique is referred to as “selective displacement”. In the “selective displacement” imprinting method, the gel coating experiments described earlier were replicated. However, in this instance, the 16 nozzles were divided into two groups. One group was composed of the four central nozzles of the array, while the second group included the surrounding nozzles. To prevent the gel buildup observed in the previous cases (Figure 4B,E,H), both groups were engaged at different rates.

### 3.5. Selective Displacement Imprinting Technique

**Figure 5:**
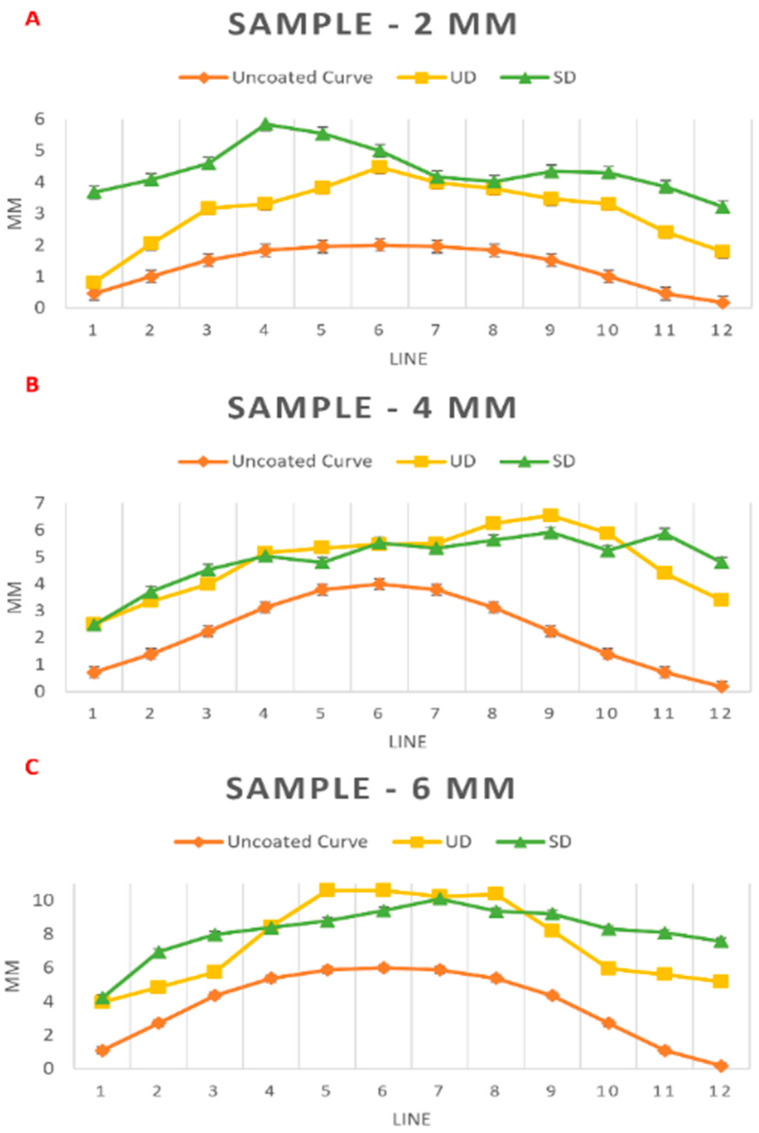
Surface contours of the testbed with gels deposited using the EP technique with uniform displacement (UD) and selective displacement (SD) deposition techniques. The plot in (**A**) corresponds to the data in Table 3 for 2 mm tall testbeds, the plot in (**B**) corresponds to the data in Table 3 for 4 mm tall testbeds, and the plot in (**C**) corresponds to the data in Table 3 for 6 mm tall testbeds. All three plots include error bars, representing the margin of error from manual measurements, with a range of +/−2 mm.

To quantify the variations in gel layer height between uniform displacement (UD) and selective displacement (SD), we measured a series of 12 vertical lines on the optical micrographs depicting side views of the samples post-gel deposition (see Figure 4). The results of these measurements are detailed in Table 3 and illustrated in Figure 5. Utilizing the data from earlier tables and applying Equation (3), we calculated the approximate height of the gel layer for each sample, as presented in Supplementary Table S4.

The outcomes of “selective displacement” deposition are shown in Figure 4C,F,I. In comparison to “uniform displacement” deposition detailed earlier, “selective deposition” yielded a similar coating for the 2 mm and 4 mm samples. However, for the 6 mm samples, the gel layer coating exhibited greater uniformity when contrasted with the “uniform displacement” method. The measured heights of the deposited layers for the “selective displacement” deposition technique are summarized in Supplementary Table S4. While the “selective displacement” technique can be advantageous for coating high-aspect-ratio features, the viscosity of the gel is critical for optimizing this process. It ensures a uniformly conformal coating across the surface, as illustrated by the excess gel deposited at the left edge of the 2 mm SD sample in Figure 4C.

## 4. Conclusions

This study showcases an advanced multi-nozzle extrusion printer designed for the conformal coating of curved surfaces. Two deposition techniques, namely “Press and Express” and “Express and Press,” have been presented. Our findings indicate that the “Express and Press” technique yields more homogeneously merged conformal layers compared to the “Press and Express” technique. We demonstrated single-nozzle deposition, 4-nozzle deposition, and gel deposition using a 16-nozzle array. Moreover, the flexibility to attach individual syringes with gel to each nozzle in the array allows for customized gel profiles. Notably, our study illustrates that employing “selective displacement” (where the four central nozzles express less gel than the remaining nozzles) achieves improved conformal gel deposition over curved surfaces compared to “uniform deposition”, where the same amount of gel is expressed from all nozzles in the array. This “selective displacement” strategy is helpful for providing coatings for steeply protruding features.

The present work demonstrates the capabilities and limitations of a novel soft matter multi-nozzle extrusion printer. The prototype was limited to a 4 × 4 array of 0.042″ nozzles. The increase in the number of nozzles and the reduction in nozzle diameter are expected to improve the conformity of the coating layers. Enhancements to the conformal extrusion printer can be achieved by employing higher density arrays of nozzles, such as 25 × 25 or 36 × 36, and utilizing more resilient adhesives and sealants to prevent system leakages observed in our current study. The elimination of leaks will allow for the experimental validation of our proposed mathematical model for deposited volumes. Although increasing the number of nozzles in the array and reducing their diameter can improve resolution, there is a limit to how much resolution can be enhanced. As nozzle size decreases, the pressure required for droplet deposition increases, which in turn raises shear stress. When depositing cell-laden gels, the shear stress must not exceed critical thresholds that could cause cell damage, thereby imposing a lower limit on nozzle size. Another limitation arises from the spacing between neighboring nozzles—sufficient gaps are needed to allow for nozzle movement and to accommodate the placement of springs. Future work is anticipated to leverage the presented extrusion 3D printer for the conformal deposition of cell-laden hydrogels on curved surfaces, including applications such as heart valve leaflets.

## Figures and Tables

**Table 1:** Approximate measurements of the gel-covered area and the deposited diameters of the three deposition samples as shown in Supplementary Figure S4A–C using the “Express and Press” technique with a 16-nozzle array and the characteristic heights of the deposited gel layers as shown in Supplementary Figure S4D–F using the “Express and Press” technique with a 16-nozzle array.

Sample	Volume Deposition (µL)	Area (mm^2^)	Longitudinal Diameter (mm)	Transverse Diameter (mm)	Average Diameter (mm)
SF4A	100	141.61	12.48	13.64	13.06
SF4B	200	339.66	20.97	18.77	19.87
SF4C	300	527.60	28.03	25.70	26.86
**Sample**	**Average Height (mm)**	**Minimum Height (mm)**		**Maximum Height (mm)**	
SF4D	3.11	2.03		3.40	
SF4E	3.85	2.09		5.14	
SF4F	7.12	5.08		9.26	

**Table 2:** Approximate volume calculations for panels A to C from Supplementary Figure S4 according to Table 1 using the “Express and Press” technique with the 16-nozzle array.

Sample	Average Diameter (mm)	Average Height (mm)	Calculated Volume (µL)	Displaced Volume (µL)	Lost Gel Volume (µL)	Deposited Volume (µL)
SF3A	13.06	3.11	416.22	1600	1183.78	416.22
SF3B	19.87	3.85	1192.64	3200	2007.36	1192.64
SF3C	26.86	7.12	4034.43	4800	765.57	4034.43

**Table 3:** Height measurements of deposited gel over 2, 4, and 6 mm tall testbeds using the “Express and Press” technique with the 16-nozzle array.

**Sample: 2 mm**												
**Line**	**1**	**2**	**3**	**4**	**5**	**6**	**7**	**8**	**9**	**10**	**11**	**12**
**Uncoated Curve (mm)**	0.46	1	1.52	1.83	1.96	2	1.96	1.83	1.52	1	0.46	0.17
**UD (mm)**	0.81	2.05	3.17	3.31	3.82	4.47	3.98	3.79	3.46	3.3	2.41	1.79
**SD (mm)**	3.68	4.08	4.59	5.84	5.54	5	4.16	4.01	4.34	4.3	3.86	3.21
**Sample: 4 mm**												
**Line**	**1**	**2**	**3**	**4**	**5**	**6**	**7**	**8**	**9**	**10**	**11**	**12**
**Uncoated Curve (mm)**	0.71	1.39	2.24	3.13	3.79	4	3.79	3.13	2.24	1.39	0.71	0.18
**UD (mm)**	2.51	3.36	3.99	5.15	5.34	5.47	5.5	6.25	6.54	5.88	4.4	3.39
**SD (mm)**	2.47	3.71	4.53	5.04	4.8	5.53	5.34	5.63	5.92	5.24	5.87	4.8
**Sample: 6 mm**												
**Line**	**1**	**2**	**3**	**4**	**5**	**6**	**7**	**8**	**9**	**10**	**11**	**12**
**Uncoated Curve (mm)**	1.09	2.71	4.35	5.37	5.87	6	5.87	5.37	4.35	2.71	1.09	0.17
**UD (mm)**	3.95	4.83	5.74	8.44	10.6	10.6	10.23	10.37	8.2	5.94	5.6	5.16
**SD (mm)**	4.21	6.94	7.97	8.39	8.78	9.39	10.09	9.36	9.21	8.3	8.09	7.57

## Data Availability

The data are available upon request.

## Supplementary Materials

The following supporting information can be downloaded at https://www.mdpi.com/article/10.3390/mi16050505/s1: Supplementary Figure S1: Droplets produced using PE and EP techniques with a single nozzle on a flat surface; Supplementary Figure S2: Droplets produced using the EP technique with a single nozzle on a flat surface; Supplementary Figure S3: Gel deposition with PE and EP techniques using a 4-nozzle array on a flat surface; Supplementary Figure S4: Droplet measurement for EP technique using a 16-nozzle array on a flat surface; Supplementary Table S1: The measured values of the grid from Supplementary Figure S1B using the “Express and Press” technique with a single nozzle; Supplementary Table S2: The measured values of the grid from Supplementary Figure S2A,D using the “Express and Press” technique with a single nozzle; Supplementary Table S3: The gel layer area corresponds to three different volume displacements using the 4-nozzle array with the “Express and Press” deposition technique; Supplementary Table S4: Layer height calculation for 2, 4, and 6 mm samples with uniform displacement (UD) and selective displacement (SD) using the “Express and Press” technique with the 16-nozzle array.
