# Supplementary material for: Characterization of the Coating Layers Deposited onto Curved Surfaces Using a Novel Multi-Nozzle Extrusion Printer
Source: Micromachines (Basel). 2025 Apr 26;16(5):505. doi: 10.3390/mi16050505 (PMC12113764; doi:10.3390/mi16050505)
Supplement: Supplementary file 1 [file micromachines-16-00505-s001.zip › micromachines-3583509-supplementary.pdf]

Supplementary Material for

Characterization of the Coating Layers Deposited onto Curved Surfaces with a Novel Multi-Nozzle Extrusion Printer

Ramses Seferino Trigo Torres [1], Lawrence Kulinsky [2,*] and Arash Kheradvar [1,*]

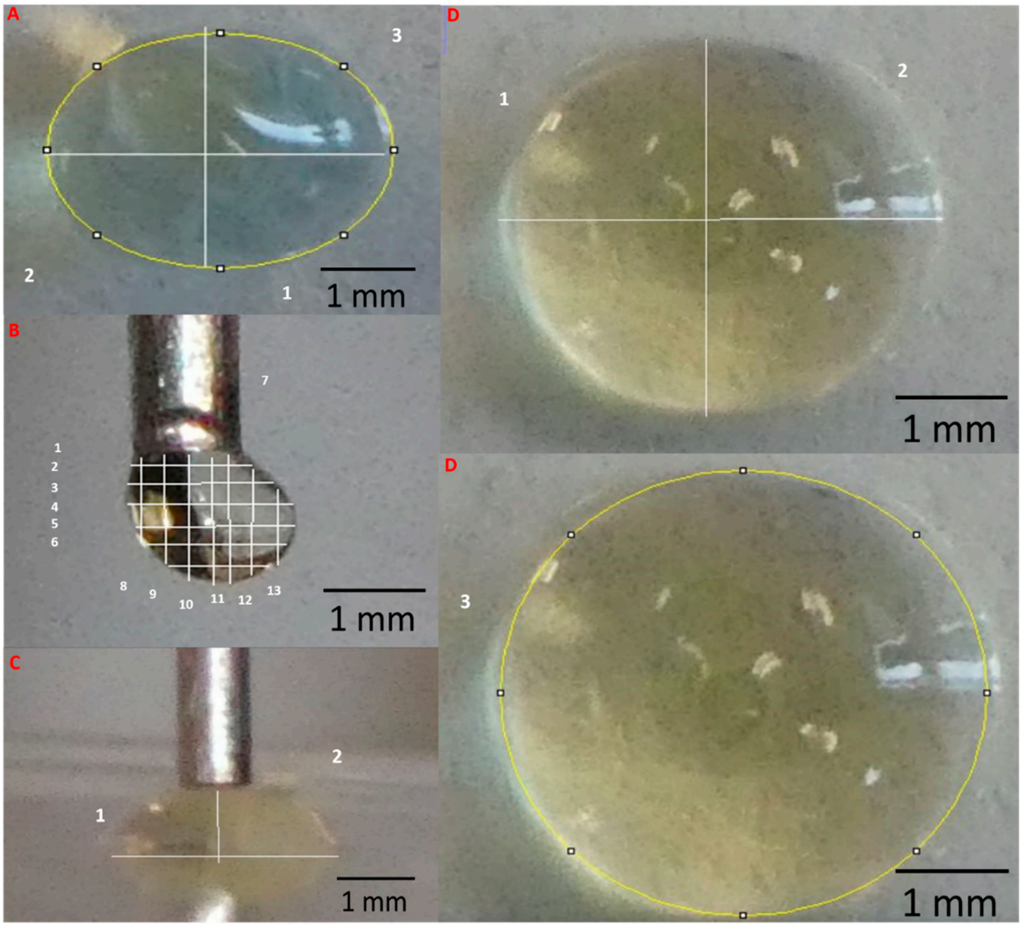

Supplemental Figure S1. Droplets Produced Using PE and EP Techniques with a Single Nozzle on a Flat Surface. (**A**) PE Technique - Top view. Items 1 and 2 in the image of the deposited gel droplet identify the major axes, while arrow 3 denotes the measured circumference used to calculate the surface area covered by the gel. (**B**) EP Technique - Side view with arrows 1 through 13 indicating respective measurements of the suspended gel droplet. These values are detailed in Table 1. (**C**) EP Technique - Side view of the gel droplet at the point of deposition onto the surface with arrows 1 and 2 representing measurements. (**D**) EP Technique - Top view of the gel droplet after deposition as the nozzle is raised and separated. Arrows 1 and 2 indicate the measured major axes, while arrow 3 signifies the measured circumference and the corresponding covered surface area.

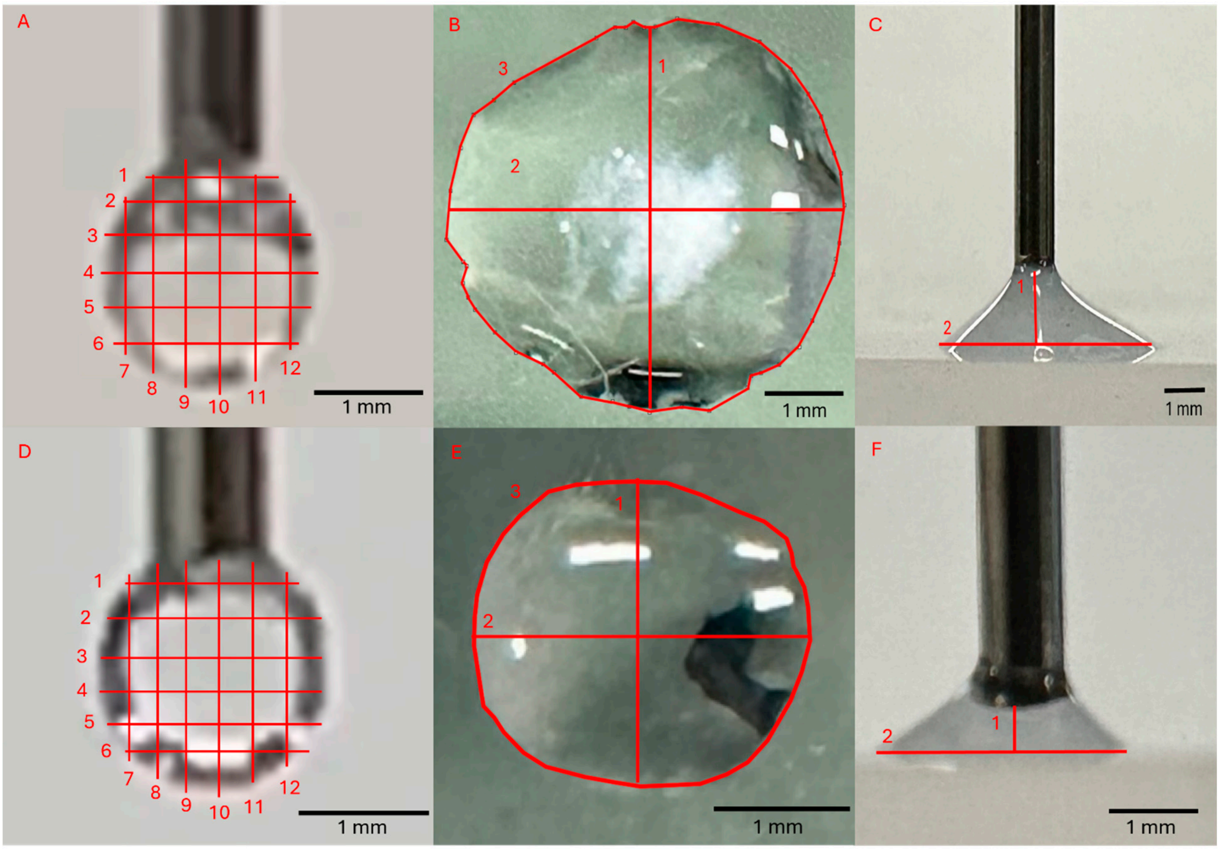

Supplemental Figure S2. Droplets Produced Using EP Technique with a Single Nozzle on a Flat Surface. (A) Side view with lines 1 through 12 indicating respective measurements of the suspended collagen droplet. These values are detailed in Supplemental Table 2. (B) Top view of the collagen droplet after deposition as the nozzle is raised and separated. Lines 1 and 2 indicate the measured major axes, while arrow 3 signifies the measured circumference and the corresponding covered surface area. (C) Side view of the collagen droplet at the point of deposition onto the surface with arrows 1 and 2 representing measurements. (D) Side view with lines 1 through 12 indicating respective measurements of the suspended hyaluronic acid droplet. These values are detailed in Supplemental Table 2. (E) Top view of the hyaluronic acid droplet after deposition as the nozzle is raised and separated. Lines 1 and 2 indicate the measured major axes, while arrow 3 signifies the measured circumference and the corresponding covered surface area. (F) Side view of the hyaluronic acid droplet at the point of deposition onto the surface with arrows 1 and 2 representing measurements.

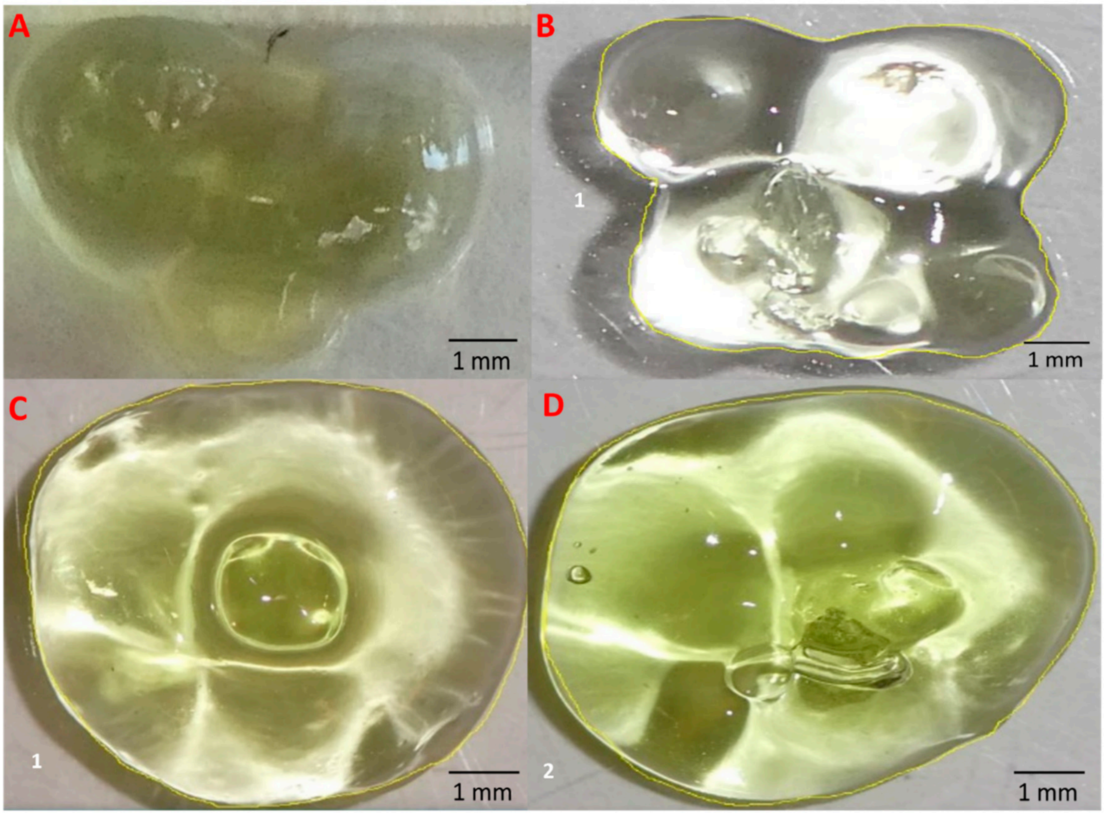

Supplemental Figure S3. Gel Deposition with PE and EP Techniques Using a 4-Nozzle Array on a Flat Surface. (**A**) Gel deposition using PE; the printed layer is compromised due to system leaks and backpressure. (**B**) Gel deposition using EP Technique with a 0.5 mm clearance and 500 µl gel deposition. A yellow line (indicated by white arrow 1) serves as a guideline for area measurement. (**C**) Gel deposition using EP Technique with a 0.5 mm clearance. Yellow contour lines (1 and 2) serve as guidelines for area measurement. (1) 1000 µl gel deposition. (2) 1500 µl gel deposition.

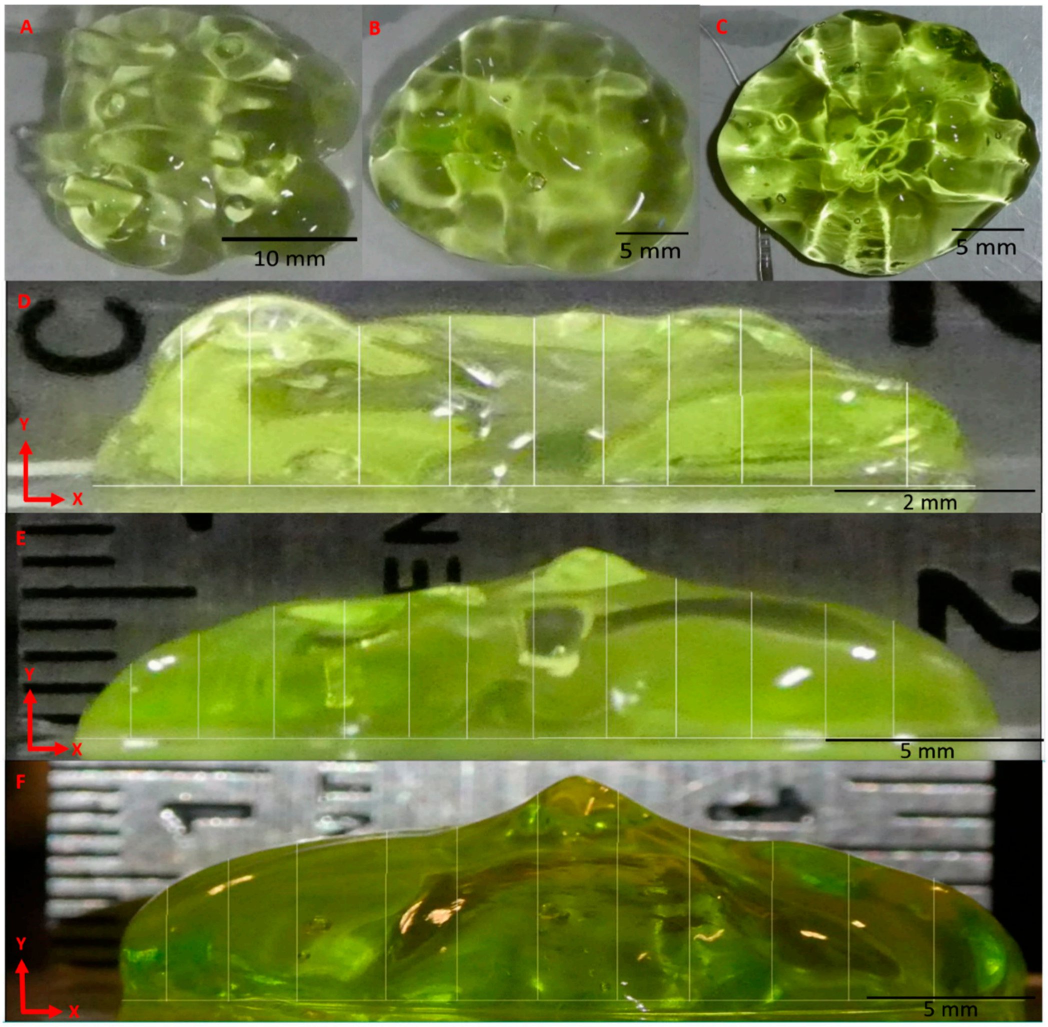

Supplemental Figure S4. Droplet Measurement for EP Technique Using a 16-Nozzle Array on a Flat Surface. (**A**) Top view of the sample with 100 µl per nozzle deposition. (**B**) Top view of the sample with 200 µl per nozzle deposition. (**C**) Top view of the sample with 300 µl per nozzle deposition. (**D**) Front view of the sample from (B) with 10 points designated for height measurement. (**E**) Front view of the sample from (C) with 10 points designated for height measurement. (**F**) Front view of the sample from (**D**) with 10 points designated for height measurement.

Supplemental Table S1. The measured values of the grid from Supplemental Figure 1B using Express and Press technique with single nozzle. SD: Standard Deviation.

**Label**	**1**	**2**	**3**	**4**	**5**	**6**	**7**
**Length (mm)**	1.18	1.42	1.57	1.55	1.36	0.97	0.94
**Label**	**8**	**9**	**10**	**11**	**12**	**13**	
**Length (mm)**	1.21	1.41	1.42	1.44	1.18	0.84	
**Label**	**Mean**	**SD**	**Min**	**Max**			
**Value (mm)**	1.24	0.25	0.84	1.57			

Supplemental Table S2. The measured values of the grid from Supplemental Figure 2A and 2D using Express and Press technique with single nozzle. SD: Standard Deviation.

**Material**	**Label**	**1**	**2**	**3**	**4**	**5**	**6**	**7**	**8**	**9**	**10**	**11**	**12**	**Mean**	**SD**
**Collagen**	**Value (mm)**	1.28	1.70	1.92	1.96	1.79	1.43	1.24	1.61	2.01	2.07	1.87	1.55	1.70	0.28
**Hyaluronic Acid**		1.41	1.72	1.76	1.77	1.60	1.04	1.00	1.50	1.66	1.69	1.63	1.10	1.49	0.29

Supplemental Table S3. The gel layer area corresponds to three different volume displacements using the 4-nozzle array with the Express and Press deposition technique.

**Gel Deposition (µl)**	**Area (mm2)**
500	21.93
1000	89.02
1500	149.80

Supplemental Table S4. Layer height calculation for 2-, 4- & 6-mm samples with uniform displacement (UD) and selective displacement (SD) using Express and Press technique with 16-nozzle array.

Height of Deposited Gel (mm)

Sample/ Line	Model	1	2	3	4	5	6	7	8	9	10	11	12
**2 mm**	**UD**	0.35	1.05	1.65	1.48	1.86	2.47	2.02	1.96	1.94	2.3	1.95	1.62
	**SD**	3.22	3.08	3.07	4.01	3.58	3	2.2	2.18	2.82	3.3	3.4	3.04
**4 mm**	**UD**	1.8	1.97	1.75	2.02	1.55	1.47	1.71	3.12	4.3	4.49	3.69	3.21
	**SD**	1.76	2.32	2.29	1.91	1.01	1.53	1.55	2.5	3.68	3.85	5.16	4.62
**6 mm**	**UD**	2.86	2.12	1.39	3.07	4.73	4.6	4.36	5	3.85	3.23	4.51	4.99
	**SD**	3.12	4.23	3.62	3.02	2.91	3.39	4.22	3.99	4.86	5.59	7	7.4
